# Trade-offs between immunity and competitive ability in fighting ant males

**DOI:** 10.1186/s12862-023-02137-7

**Published:** 2023-08-07

**Authors:** Sina Metzler, Jessica Kirchner, Anna V Grasse, Sylvia Cremer

**Affiliations:** ISTA (Institute of Science and Technology Austria), Am Campus 1, Klosterneuburg, 3400 Austria

**Keywords:** Male-male competition, Male fighting, Immunity-reproduction trade-off, Immune gene expression, Social immunity, Inclusive sexual selection, Social insects, *Cardiocondyla* ants, *Metarhizium* fungus

## Abstract

**Background:**

Fighting disease while fighting rivals exposes males to constraints and trade-offs during male-male competition. We here tested how both the stage and intensity of infection with the fungal pathogen *Metarhizium robertsii* interfere with fighting success in *Cardiocondyla obscurior* ant males. Males of this species have evolved long lifespans during which they can gain many matings with the young queens of the colony, if successful in male-male competition. Since male fights occur inside the colony, the outcome of male-male competition can further be biased by interference of the colony’s worker force.

**Results:**

We found that severe, but not yet mild, infection strongly impaired male fighting success. In late-stage infection, this could be attributed to worker aggression directed towards the infected rather than the healthy male and an already very high male morbidity even in the absence of fighting. Shortly after pathogen exposure, however, male mortality was particularly increased during combat. Since these males mounted a strong immune response, their reduced fighting success suggests a trade-off between immune investment and competitive ability already early in the infection. Even if the males themselves showed no difference in the number of attacks they raised against their healthy rivals across infection stages and levels, severely infected males were thus losing in male-male competition from an early stage of infection on.

**Conclusions:**

Males of the ant *C. obscurior* have a well-developed immune system that raises a strong immune response very fast after fungal exposure. This allows them to cope with mild pathogen exposures without compromising their success in male-male competition, and hence to gain multiple mating opportunities with the emerging virgin queens of the colony. Under severe infection, however, they are weak fighters and rarely survive a combat already at early infection when raising an immune response, as well as at progressed infection, when they are morbid and preferentially targeted by worker aggression. Workers thereby remove males that pose a future disease threat by biasing male-male competition. Our study thus reveals a novel social immunity mechanism how social insect workers protect the colony against disease risk.

**Supplementary Information:**

The online version contains supplementary material available at 10.1186/s12862-023-02137-7.

## Background

Escalated male fighting over access to females is common among many vertebrate species [1], and can also occur in invertebrates, like cephalopods [2], butterflies [3], parasitoid and fig wasps [4, 5], as well as some ant species [6–11]. Despite the high costs that fights can bear for both competitors in terms of e.g. time consumption and risk of injury, even lethal combats evolve when it allows the winning males to monopolize mating with the females [1, 12].

Male fighting ability relies on physical strength, which often becomes compromised when males get sick. Mounting an immune response to fight an infection requires resources that cannot simultaneously be invested into fighting rivals, leading to a classic trade-off situation. Under infection, reallocation of resources away from growth and reproduction is therefore commonly observed already at early stages of infection like the incubation period before typical disease symptoms appear [13–15]. When disease has progressed, hosts often even show extensive “sickness behaviours” like lethargy and disengagement from most social interactions [16–20], thereby conserving energy to fight off the infection [21–24]. However, refraining oneself from engaging in male-male competition when sick does not seem to be a good strategy for males that will not gain a later chance for reproduction. This is the case either when disease will end deadly, or when hosts are so short-lived that reproduction cannot be postponed to after they have overcome the disease [25]. Under such conditions, males have nothing to lose and often even fight more violently against a rival, as a “terminal investment” strategy [26]. Due to these apparent trade-offs, short-lived species typically do not invest many resources into a functional immune system, and in longer-lived species, immunity is often reduced during the reproductive period (also due to a direct immunosuppressive effect of the male hormone testosterone) [16–20]. Moreover, increased irritability and aggression are common behavioural reactions to disease in vertebrates, including humans [21, 27], which can also be found in insects, including ants [28]. Therefore, it is not straightforward to predict the effect of infection on male fighting ability, particularly since it will also be affected by (i) the level of infection, i.e. if it represents a low-level infection that causes mild or no disease symptoms, or a high-level infection inducing severe disease and death, and (ii) on its stage after exposure, i.e. whether the male suffers from progressed infection or is still in the early incubation period mounting up its immune response.

When fighting occurs in a social context, it may be further influenced by conspecifics. For example, the workers of social insects would have the potential to bias the outcome of male-male competition, if it takes place inside the colony. Such intra-nest fighting has evolved in several ant species in the genera *Cardiocondyla* and *Hypoponera*, when mating occurs in the maternal nest and emerging queen numbers are small enough to be monopolized by the winner male [6–8, 10, 12, 29]. Whilst it is not yet known if such worker manipulation of male fights exists, it was found that ant workers can affect sexual selection by choosing or preferring some males as the queen’s partners [30–32]. As workers are sterile but gain inclusive fitness via the reproduction of their related queens and males, such interference with mate choice has been referred to as “inclusive sexual selection” [31]. Biasing the outcome of male-male competition would similarly allow workers to favour some males over others as the queens’ future mates. In addition, worker aggression towards infected males could represent a form of social immunity [33]. By helping the healthy male to win the fight, the diseased rival, who poses an infection risk to the colony, would be eliminated, reducing the danger of a disease outbreak in the colony. Such bias introduced by workers on male-male competition would therefore correspond to an ‘inclusive form’ of the Hamilton-Zuk hypothesis of parasite-mediated sexual selection [19, 34].

We used the ant *Cardiocondyla obscurior* as a model system to test for the occurrence of worker interference with male-male competition in ants, and its possible interplay with male disease state. As typical for invasive ants [35–37], the queens and males of *C. obscurior* mate inside the nest [8, 29, 38]. The wingless fighter males patrol the nest to identify emerging virgin queens to mate with, as well as emerging rival males to attack and engage in lethal combats. During a fight, males grab and besmear the rival male with a hindgut secretion that attracts workers, which then bite the besmeared male to death [39, 40]. Besmearing is typically performed by the older and hence dominant male, yet fights between similarly-aged rivals often involve mutual grabbing and besmearing attacks [40], which will give workers the opportunity to bias the fight outcome.

Successful males that are able to quickly detect emerging rivals thus can gain access to virgin queens over a long period of multiple weeks and can perform up to 50 and more matings [41]. This has led to the evolution of both, a prolonged lifespan comparable to a worker’s lifespan, as well as a lifelong spermatogenesis in these fighter males [8, 42]. *C. obscurior* fighter males therefore greatly differ from ‘typical’ ant males, which are very short-lived, have completed their spermatogenesis before adult emergence and only have a single mating opportunity per lifetime in a mating flight, during which they engage in scramble competition with the other males [9, 12, 43]. We would thus expect *C. obscurior* males to invest in a functioning immune system – in contrast to other social insect males with reported low immunocompetence [44–46]. Yet, this could make them vulnerable to an immunity-reproduction trade-off.

We therefore observed how exposure of *C. obscurior* males to the fungal pathogen *Metarhizium robertsii* – a common natural fungal pathogen of ants [47–49] – affects the male’s own fighting ability and the workers’ interference in fights between a healthy and an infected male. To test for a possible trade-off between immunity and fighting ability we either exposed males to a low or a high dose of the fungal pathogen. Since the outcome of *Metarhizium* infections are dose-dependent [50, 51], the low dose was chosen to induce only a mild infection that the male’s immune system was expected to be able to cope with, whilst the high dose should in most cases cause severe disease, leading to death. For both, low and high dose, we tested the fighting success of infected males when in a combat situation with a healthy male (i) early in the infection, i.e. directly after exposure, when the pathogen’s infectious stages (the conidiospores, hereafter abbreviated as spores) start penetrating the host’s cuticle, and (ii) at a progressed infection stage two days after exposure [52]. We expected that not only severe disease but already mounting an early immune response may negatively impact the males’ fighting ability. Apart from that, since the males face both a deadly disease and a deadly combat situation, they should show terminal investment of all their (remaining) resources into fighting. We also predicted that – if workers would indeed bias fight outcome – they should discriminate against the infected male, either to prevent pathogen spread by contagious males (early after exposure) or because the males represent a future infection risk to the colony by developing into a sporulating cadaver (when fighting at later stage of disease).

## Results

### Severe infections reduce male fighting success from an early disease stage

**Fig. 1:**
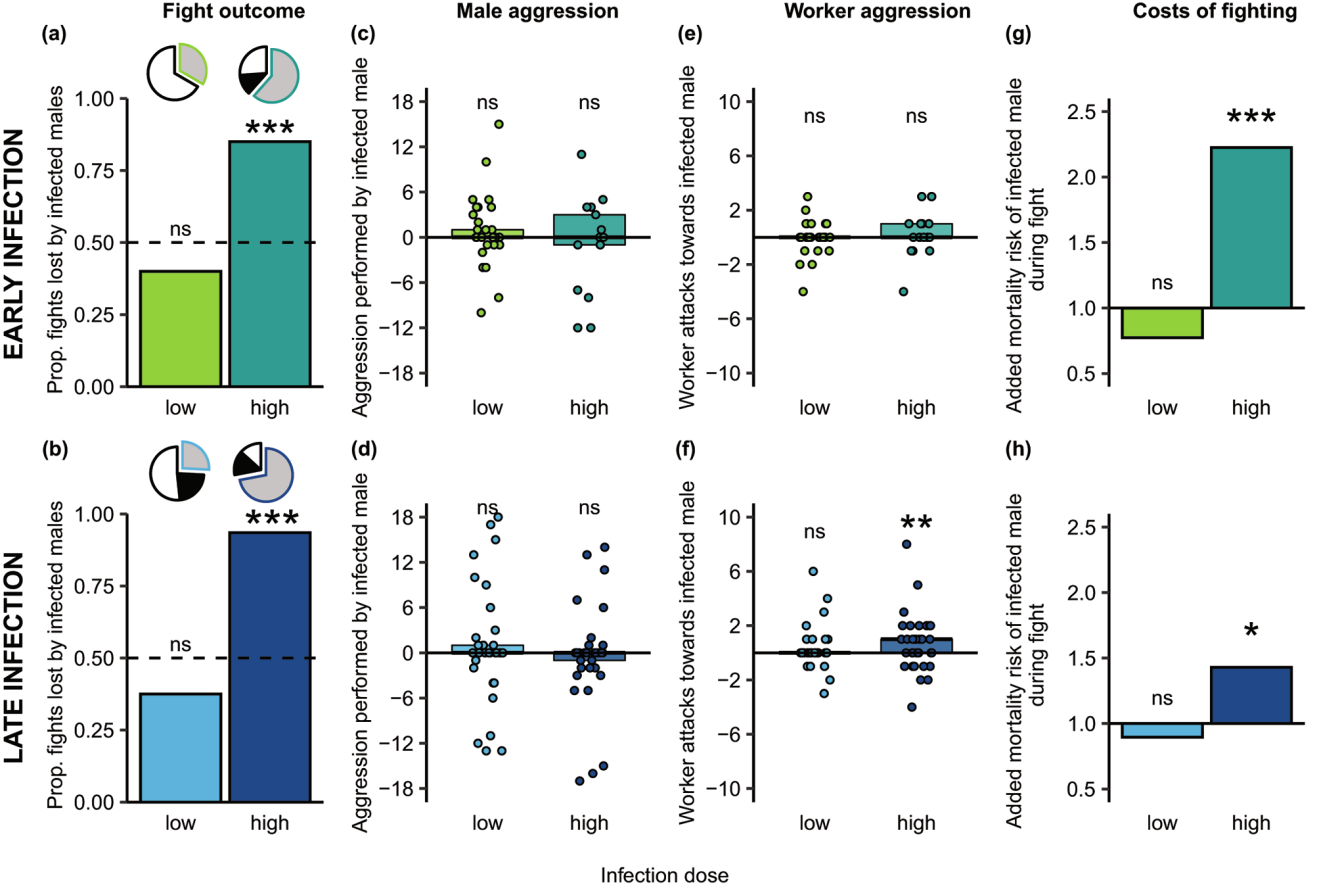
Fighting ability of infected males in dependence of infection dose and stage. Fight outcome for infected males compared to their healthy rival males at (**a**) the early (green) and (**b**) the late (blue) stage of infection. Pie charts show the proportion of fights that ended undecided with either both males still alive (white), or both males dead (black), or decided with one clear winner and loser (grey; Table S2). In the decided fights (grey offset slice with coloured outline), infected males had a higher risk of losing the fight than by chance (dotted line) after exposure to a high, but not low pathogen dose (bars depict the proportion of fights lost by the infected male; darker tone reflects higher dose; based on a total of 347 fights, detailed in Table S1). The aggression level of infected males (**c,d**) did not differ from that of their healthy rivals across infection stage or level, whilst workers showed increased aggression only to late-stage high-level infected males (**e,f**). Individual data points depict aggression by (c,d) and towards (e,f) each infected male compared to its healthy rival (aggression_infected−healthy_, i.e. resulting in a zero value under equal aggression and positive values for higher aggression), boxes give the 95% CI and median as black line (based on 112 observed fights between healthy and infected males). The added risk of dying for infected males when fighting (**g,h**) compared to their baseline mortality when non-fighting during the same period (Fig. S1; Table S3) was significantly increased for males in the early and late stage of a high-level infection, where their mortality risk during fight increased 2.2-fold at the early stage and 1.4-fold at the late-stage. Bars depict the fold change of mortality during combat to the males’ respective baseline mortality (value of 1 depicts equal mortality). Based on 201 fights. Significant deviation from chance (50:50) (a,b), from the healthy male (c-f) and from their non-fighting baseline mortality (g,h) given for each group. * p < 0.05, *** p < 0.001, ns = non-significant

*C. obscurior* males that had been exposed to a high pathogen load of *M. robertsii* had a reduced fighting success, when in a 24 h-long combat situation with a healthy male (exposed to a pathogen-free sham treatment; control male). This was equally so for the early stage of infection, when males were put into the fight situation immediately after pathogen exposure (Fig. 1a), and when their infection had already progressed to a later stage, as males had been exposed already 48 h before the start of the fight (Fig. 1b). Over all 347 fights (Table S1), we observed three different fight outcomes after 24 h. These were that fights either remained undecided, with both males either being alive or both males being dead, or that they ended as ‘decided fights’ with one rival surviving and the other being killed in combat (for the frequency of these outcomes see pie charts in Fig. 1a,b; for statistical comparisons see Table S2). Notably, compared to fights between two healthy males, fights, in which one of the males was infected with a high dose, more frequently ended with one clear winner and one clear loser (trending in early infection: GLMM, p = 0.059; significant in late infection: p = 0.039; for statistical details see Table S2). Importantly, in the great majority of these decided fights, it was the infected male that lost the competition, independent of the stage of its infection: 85% (34/40) of the early-infected males (Chi-Square test, χ^2^ = 11.169, df = 1, p < 0.001), and 93.5% (43/46) of the late-infected males (Chi-Square test, χ^2^ = 21.445 df = 1, p < 0.001) had been killed at the end of the fighting period when exposed to a high pathogen dose (Fig. 1a,b).

When males had only received a low pathogen dose that rarely causes disease, there was no difference between the probability of the fight ending with a clear winner and loser compared to fights between two healthy males, for both infection stages (early: GLMM, p = 0.236; late: p = 0.433; for statistical details see Table S2). Moreover, when fights were decided, low level-infected males did not lose the fights with a higher probability than their healthy rivals (Fisher’s Exact test, early: 4/10, p = 1.000; late: 3/8, p = 1.000; Fig. 1a,b; see also Table S2).

### Workers attack severely-infected males at a late disease stage

To test whether the fighting success of infected males could be explained by their own aggression level towards their competitor, or by the aggression they received from the workers, we observed the behaviour of the fighting males and their workers. We found that – independent of dose and stage of infection – infected males showed the same level of aggression towards their rivals as the healthy males did towards them (Fig. 1c,d; GLMMs,

early infection low dose: χ^2^ = 0.459, p = 0.853 and high dose: χ^2^ = 0.073, p = 0.787, late infection low dose: χ^2^ = 0.261, p = 0.609 and high dose: χ^2^ = 0.178, p = 0.673; all df = 1). This means that even the high-level infected males, who finally lost the fight in the majority of cases (Fig. 1a,b), did not perform fewer attacks. The number of worker attacks towards the infected male compared to its healthy rival was twice as high in fights with high-level exposed males at a late stage of their infection (GLMM, χ^2^ = 8.155, df = 1, p = 0.009), but not in the early stage (GLMM, χ^2^ = 1.516, df = 1, p = 0.436), or after low-dose exposure of the males (Fig. 1e,f; GLMMs, early low: χ^2^ = 0.035, df = 1, p = 0.853; late low: χ^2^ = 2.599, df = 1, p = 0.214).

### Males suffer highest fighting costs early in severe infection

Worker aggression could explain why high-level infected males at a late disease stage frequently lost the fights. However, at the early infection stage, the high-level infected males had equally low fighting success, even if workers did not treat them with increased aggression (Fig. 1a,b,e,f). We therefore determined the baseline mortality risk of the males in their respective fighting period in the absence of a fight, to disentangle, how much of the mortality of fighting males could be contributed to the fight itself, and how much to an already impaired survival due to infection. To this end, individual males were kept in the absence of a competitor with their workers for a 24 h period, starting either directly after pathogen exposure, or after infection had been established for 48 h. Whilst healthy males had a mortality risk of 8–9% (Table S3), infection in general increased this risk. At the early stage of infection, this was only significant for males exposed to a high pathogen dose (low dose: 17%; GLMM, χ^2^ = 3.323, df = 1, p = 0.091; high dose: 29%; GLMM, χ^2^ = 8.992, df = 1, p = 0.005). When infection had progressed, the males’ baseline mortality risk in the absence of fighting was already pronounced at the low dose (36%; GLMM, χ^2^ = 7.624, df = 1, p = 0.010), and reached high morbidity (56% mortality) at the high pathogen dose (GLMM, χ^2^ = 24.820, df = 1, p < 0.001; Fig. S1; Table S3). Notably, this means that progressed infection led to a high infection-induced baseline mortality, with no significant further increase of mortality caused by fighting at the low dose (Chi-Square test, χ^2^ = 0.086, df = 1, p = 0.769). At the high dose, however, a 1.4-fold increased mortality was observed (Chi-Square test, χ^2^ = 7.107, df = 1, p = 0.010, Fig. 1h). Early-infected males showed comparably lower baseline mortality, but died more than two times more frequently during fighting when infected with a high dose (2.2-fold increased mortality; Chi-Square test, χ^2^ = 16.120, df = 1, p < 0.001; Fig. 1g, but not at the low dose; Fisher’s Exact test, df = 1, p = 0.730). Fighting costs therefore incurred for males in the early and late stage of severe infection, but were much more pronounced for early stage-infected males (Fig. 1g,h).

### Severe infection induces a strong early immune response

**Fig. 2:**
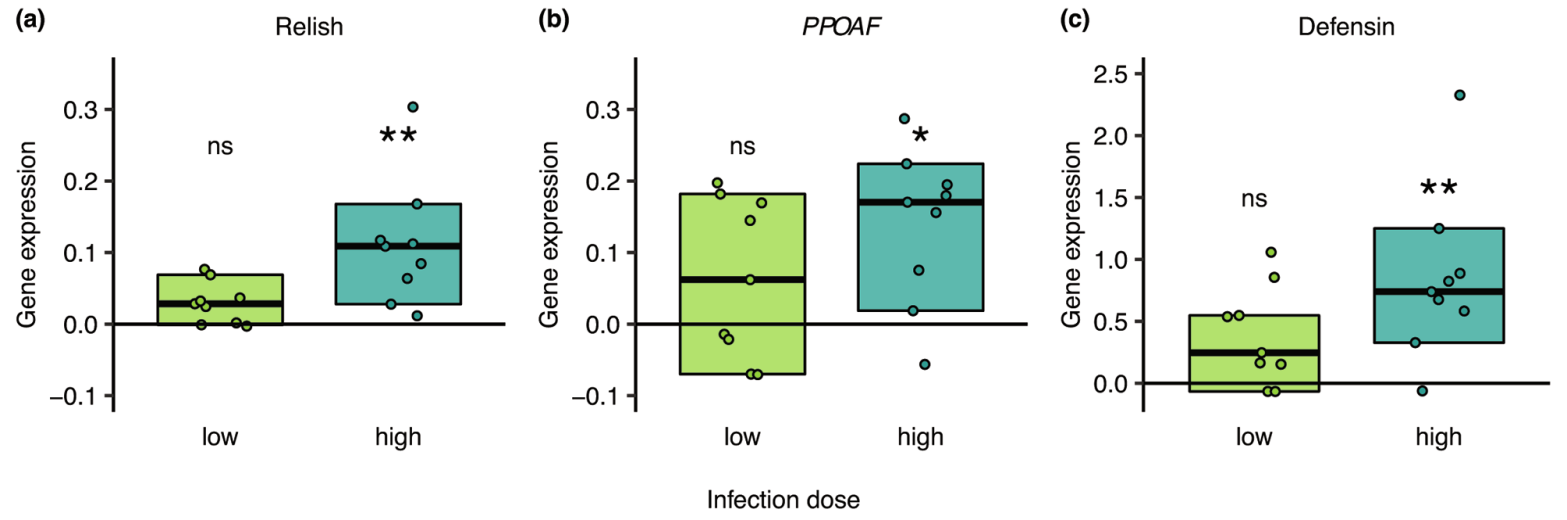
Immune gene expression 24 h after exposure to the low or high pathogen dose. Expression of all three measured immune genes (**a**) Relish, (**b**) *PPOAF*, and (**c**) Defensin was increased in infected males compared to the baseline expression level in healthy males (zero baseline) for males after high-dose (dark green), but not the low-dose (light green) pathogen exposure. For each infected male we show the relative expression level of each gene normalised to the housekeeping gene *EF1A*, relative to the median gene expression of the respective healthy control males (zero line) as individual data point, median per group as black line and 95% CI as box. Based on a total of 28 males (N = 10 control males, 9 low-dose and 9 high-dose exposed). Significant deviation to the healthy males for each dose and gene indicated by * p < 0.05, ** p < 0.01, ns = non-significant. See Fig. S2 for gene expression 12 h after exposure

These data suggest that fighting as an additional stressor may unveil costs of mounting an immune response at the early stage of infection, causing increased mortality when the immune response is strong, as expected under severe but not yet mild infection. We therefore tested if immune genes were activated already within the first 24 h after pathogen exposure, and whether this immune activation was higher after high-level exposure. Increased immune gene expression at the level of receptors and effectors would indicate that the infectious fungal spores were able to breach the host cuticle, cause infection and activate the host’s immunity cascade within 24 h after exposure. We therefore measured the expression of three genes that act at different stages of the immune response, (i) Relish (*Rel*), a signal cascade gene in the immune deficiency (IMD) pathway [53, 54]; (ii) Prophenoloxidase-activating factor (*PPOAF*), which starts the process of pathogen melanisation [55–57]; and (iii) Defensin (*Def*), an antimicrobial peptide destroying fungal pathogens [58, 59]. We indeed found that all three genes were significantly upregulated 24 h, but – with the exception of Relish (Fig. S2) – not yet 12 h after exposure to the high pathogen level (Wilcoxon-tests; high dose 12 h: *Rel*: W = 77, p = 0.045; *PPOAF*: W = 64, p = 0.267; *Def*: W = 65, p = 0.267; high dose 24 h: *Rel*: W = 80, p = 0.009; *PPOAF*: W = 75, p = 0.027; *Def*: W = 80, p = 0.009; Fig. 2, S2).

In the low-dose exposure, none of the genes was significantly upregulated compared to the baseline gene expression in healthy males (Wilcoxon-tests; low dose 12 h: *Rel*: W = 58, p = 0.473; *PPOAF*: W = 49, p = 0.780; *Def*: W = 50, p = 0.780; low dose 24 h: *Rel*: W = 65, p = 0.135; *PPOAF*: W = 50, p = 0.720; *Def*: W = 68, p = 0.098; Fig. 2, S2). Our data show that *C. obscurior* fighter males are able to upregulate the expression of their antifungal immune genes > 10-fold within 24 h and therefore show a high immune investment. Mounting such a strong immune response early after exposure could explain the observed survival costs of the males, particularly when under the additional stress of combat.

## Discussion

We found that the competitive ability of fighting *C. obscurior* males was strongly impacted by severe (but not by mild) infection, and that these negative effects already occurred early upon exposure. This is in line with general observations across vertebrates and invertebrate species that both incipient and progressed infection can be costly [13, 14] for males and negatively affect their fighting ability.

High-level infected males were inferior to their healthy rivals and suffered a very high risk of losing the combat and dying, both when entering the fight immediately after exposure to infectious *M. robertsii* spores, and with an already established infection. At this late infection stage, male morbidity was already quite substantial, since > 50% of the males would not have survived the day even without a fight. However, this was not yet the case during early infection, where male mortality risk more than doubled when confronted with a rival compared to when being kept without an opponent. This suggests a classic fighting-immunity trade-off [14] during early infection, when *C. obscurior* males strongly invest into raising an immune response. Mounting an immune response is an energetically costly process, involving transcriptional changes in both, immune and metabolic pathways (triggered e.g. by metabolic reprogramming of immune cells) and leading to highly increased glucose consumption [60, 61]. In insects, the link between immunity and metabolism becomes very obvious through the dual role of the fat body, which functions both as a metabolic organ (with an important role in energy storage and metabolic homeostasis [62]) and as key immune organ (producing immune effectors [55]). Even individual proteins play such dual roles, like e.g. the Myocyte Enhancer Factor-2 (*Mef2*), which, in its phosphorylated state activates gene expression required for anabolic processes. In contrast infection-induced dephosphorylation of *Mef2* reduces anabolic processes, but activates the expression of immune effectors like antimicrobial peptides (AMPs) [63]. Similarly, Apolipophorin III (*Apo-III*), facilitates lipid transfer [64], yet also acts as bacterial and fungal pattern recognition protein and stimulates the host’s encapsulation response [65–67]. This interwoven nature of immune pathways and metabolic processes seems to build the basis that infection-induced processes compete for energy allocation with other energy-consuming processes, like growth, energy storage and reproduction [60, 68], which may also explain why immune-challenged individuals can cope less well than healthy individuals with stressful conditions like predator attack [69]. Also in our study, the costs of the males’ immune investment became aggravated when they faced the additional stress of fighting against a rival. Mild infection, on the other hand, resulted in only a minor (non-significant) immune gene upregulation, and males were not inferior in fights against healthy rivals. This is further evidence that resource depletion by mounting an immune response may cause the observed lower competitive success of males when fighting a severe infection.

Males of the social insects, including the social bees, wasps and ants, are not generally expected to be able to raise such a strong immune response as we here describe for *Cardiocondyla* males (Fig. 2, S2), In fact, studies comparing the immunocompetence between castes found that social insect males have much lower immunocompetence than the colony’s workers or queens [44–46]. This is attributed to both genetic and life history effects, as, first, males of haplodiploid species are the haploid sex with lower variability on disease defence loci [70, 71], and, second, their lifespans are often only a few days, where even a good immune defence would not substantially increase life expectancy [72]. The observed high investment into immune defence likely evolved in *C. obscurior* fighter males due to their uncommonly long lifespan of up to several weeks or even months [41], during which they can engage in multiple (up to approx. 50) matings with the emerging virgin queens [41]. A functioning immune system may therefore be critical for males to ensure surviving at least mild pathogen threats at low cost to their fighting ability and giving them access to many more future mating opportunities. Therefore, we suggest that, even if a high-level infection (which will typically later develop into a fatal disease) induces a trade-off with the male’s fighting ability already at an early infection stage, the benefit of having a functional immune system will outweigh these costs as it allows the males to overcome mild infections without making them inferior competitors. Multiple factors, such as (i) these males not leaving their maternal nest, (ii) *C. obscurior* ants nesting in arboreal cavities rather than in pathogen-rich soil [29], and (iii) their active performance of hygiene and infection removal behaviours [73], combine to make it likely that contamination of the males with a low pathogen level either via the nest or contagious colony members will be a more common scenario in natural colonies than infection of males with very high doses.

Interestingly, infection levels did not directly impair male aggression, as we could not detect any differences in the aggressive behaviours performed by infected males at any stage or intensity of infection compared to their healthy rivals. This indicates that even the moribund males at the late stage of a severe infection recruited their last resources into fighting their competitors, likely as a terminal investment strategy [26]. However, these males were more aggressed by workers than their healthy rivals, revealing that workers engaged differently into the fights depending on the health state of the competing males. This is in line with inclusive sexual selection [30–32], in which workers bias the outcome of male-male competition. A non-exclusive explanation could be that workers attack the fatally-infected males to eliminate them before they die and infectious spores grow out of their cadavers. Such “destructive disinfection” [74] represents an efficient social immunity measure in social insect colonies [33, 75], preventing the development of sporulating cadavers that would otherwise pose a very high risk for disease outbreak in the colony [51, 74].

Workers did not discriminate against males directly after exposure, even if these males carried not yet firmly-attached infectious spores that we found to cross-contaminate the rival male in 45% (9/20) of the fights between high-level exposed and healthy males (N = 20 early stage high load fights analysed for spore transfer). However, this was also not to be expected as aggression in social immunity rarely occurs at the very early stage of pathogen exposure, but rather when infection has established [74]. Even though the males had very close contact during the fights, the contaminated rival carried on average less than 5% of the spore amount of the originally exposed male (1000 vs. 21,000 spores; see Dryad data), which its immune system was likely able to cope with.

## Conclusions

Our study revealed that disease negatively impacts male competitive ability in fighting ant males. Interestingly, however, we found that the same outcome – losing the fight – had different causes during the early and late stages of infection. While late-stage males were already moribund at the time of combat and received increased aggression by workers, early-infected males invested heavily into mounting an immune response, which translated into a high mortality when they had to additionally fight a competitor male. Whilst this was true for severe infections, mild infections only led to a non-significant immune activation and did not induce any substantial fighting costs. This shows that the fighting–immunity trade-off is dose-dependent and only occurs at high infection levels, while a well-functioning immune system likely allows males to cope with low-level pathogen exposures in the nest without compromising their fighting ability and allowing future mating success. As being successful in fighting off rivals is key for the males to gain access to female mates, males invested all their energy into fighting, even at terminal disease stages. Under this – and only under this – condition, workers took side and over-proportionally attacked the already moribund male. This inclusive sexual selection by biasing the outcome of male-male competition represents a novel mechanism allowing workers to ensure colony health through social immunity.

## Methods

### Ant hosts

Colonies of the Myrmicine ant *Cardiocondyla obscurior* (Wheeler, 1929) were collected in 2013 and 2017 from Tenerife (28.390348 N, -16.596098 E; permits ABSCH-IRCC-ES-237603 ESNC2, Expte: AFF 199/17). 12 stock colonies were reared at constant 27 °C and a day/night light cycle with 14 h of light in the laboratory, and fed twice a week with minced cockroach and were supplied with water and 30% sucrose *ad libitum*. All experiments were performed at 27 °C. Ant collection, rearing and experimental work was in line with European law and institutional guidelines.

This species shows a male diphenism of wingless fighter males that regularly occur in the nest and engage in deadly fights [6–8, 40], as well as peaceful winged disperser males that are produced under stress conditions [76]. Since only the wingless males engage in fighting, we focused on these males in our study. Males were collected as pigmented ‘ready-to-hatch’ pupae from the brood pile and kept with two nurse workers (to help eclosion), until all males were between three and six days old, and their cuticle fully sclerotised when they entered the experiment. We used these age-controlled males to avoid differences between them in their *a priori* fighting ability, as otherwise old males have a clear fighting benefit over males younger than two days of age, which still possess a soft cuticle [40]. We also size-matched the two rivals per fight, to avoid large body size differences.

### Fungal pathogen

As a pathogen, we used the fungus *Metarhizium robertsii* (strain ARSEF 2575) with an integrated red fluorescent (mRFP1) label [77], obtained from M. Bidochka, Brock University. *Metarhizium* fungi, incl. *M. robertsii*, are generalist insect pathogens that cause natural infections in ants [47–49]. Their infectious stages, the conidiospores (abbreviated as “spores” throughout), attach to the body surface of their insect hosts, germinate and breach the host cuticle to cause internal infection shortly after. High infection doses cause host death within several days, followed by formation of new infectious particles from the sporulating cadaver [52, 78]. Low-level infections rarely cause disease, and can even induce immune stimulation protecting against future infection [50, 79].

Prior to each experiment, spore suspensions were taken from long-term storage (–80 °C) and cultivated on sabouraud dextrose agar (SDA) at 23 °C for approximately three weeks. Conidiospores were harvested in 0.05% Triton X 100 (Sigma-Aldrich; in autoclaved distilled water). Spore suspensions were counted with a Cellometer Auto M10 counter (Nexcelom) and adjusted to the respective concentrations needed for the experiment (see below). The germination rate of spore suspensions was confirmed to be > 95% before the start of each experiment. To induce infection, males were briefly dipped into the spore suspension and placed on filter paper to absorb excess liquid. Healthy control males were set up in parallel to the fungal treatment in each experiment. They were treated the same way, except that they were only dipped into 0.05% Triton X (sham treatment).

### Experimental setup

To determine the effect of early infection, males were dipped into a suspension of either 10^6^ spores/ml (low dose) or 10^9^ spores/ml (high dose), and were placed with five workers (randomly chosen from the same stock colony from outside the brood chamber) and a second male from their colony, all sham-treated. For both the low and high dose, we equally set up control fights, where both males had only received the sham treatment (total 168 early-stage fights, detailed in Table S1). Workers immediately groom off spores from freshly-exposed colony members, so that the number of infectious spores that will then enter the body of the exposed host is drastically reduced [80–82]. As spores on a freshly-exposed male are still removable, it is also possible that they cross-contaminate their nestmates [50], including the rival male, during this period.

To obtain late infection stages, we therefore kept each male in isolation for 48 h post exposure before placing them to the workers and rival male. During this period, the spores firmly attach and enter the body to cause internal infection [52], so that males also no longer carry any infectious spores on their body [80] when entering the fight. To prevent these males developing multi-fold higher infection loads due to the lack of spore removal by grooming workers, we used lower exposure doses for induction of these late-stage infections (low dose: 10^4^ spores/ml; high dose: 10^6^ spores/ml). Sham-treated control males were similarly isolated for 48 h, and the workers kept for 48 h in groups of 35 individuals until the start of the fight (total 179 late-stage fights, detailed in Table S1). All fights contained the two rival males and five workers and were performed in experimental containers (height 28 mm, diameter 25 mm) with a plastered and watered base to ascertain the required humidity. All ants per fight replicate (males and workers) originated from the same colony (with 12 different colonies being used as source). The fight outcome was determined 24 h after putting the two males together and with their workers, afterwards all ants were frozen.

We determined the fight outcome, i.e. if the combat remained undecided (both males alive or both males dead), or had a clear winner (the surviving male) and loser (the dead male; ‘decided fights’) for all 347 early and late stage, low and high dose, as well as control fights (see Table S1). 97 out of the 236 high-dose fights and all 111 low-dose fights were further observed for detailed behavioural interactions between the two males and the workers towards the males, for which both males were individually colour-coded before the fight (total N = 208 observed fights). 139 high-dose fights were analysed only for fight outcome, so that males remained unmarked during the fight (with the exception of 12 fights in which males were colour-coded but only fight outcome was analysed). Of these 127 unmarked high-load fight setups, 50 were control fights and 77 contained one infected and one healthy male. 48 of the latter (20 early stage and 28 late stage fights) had a clear winner and a clear looser (i.e. were ‘decided’). To determine, whether the infected males were the winners or losers of these fights, we determined the spore load of both males by PCR at the end of the experiment, and could reliably distinguish between the two males in 40/48 cases, as well as determine the cross-contamination load for the healthy male (see detailed below). Note that the PCR method is not sensitive enough to distinguish low-level infected males from their healthy rivals, which is why all low-load fights were performed with colour-coded males (in addition to all high-load fights, for which we performed behavioural observations, see Table S1). For colour-coding, we applied a tiny dot of metallic email colour (Revell) on the male’s dorsal abdomen (gaster) using a micro dissection needle holder with a fine tungsten needle (1 μm tip diameter), and let the colour dry for multiple hours before start of the experiment. Note that during observation it was unknown to the observer, which colour reflected which male treatment). As per treatment group the proportion of infected individuals being the loser of a fight did not differ between male identification method (PCR vs. colour), we (i) concluded that colour-coding of the males did not alter fight outcome, and (ii) pooled the two methods for analysis of who was the winner or loser in the decided fights. In total, 112 fights ended decided, yet since the PCR method was unable to provide a reliable distinction in eight fights (see above), we could determine the identity of the healthy vs. infected male winning or losing for 104 of the decided fights.

### Spore load quantification by ddPCR

In 48 fights of differently-treated males with one male being dead and the other alive, we determined whether it was the infected or the healthy male that had lost the competition. To this end, we froze both rivals after the fight, extracted DNA and ran a droplet digital PCR (ddPCR; Bio-Rad) for direct quantification of their spore load.

For DNA extraction, the samples were homogenized using a TissueLyser II (Qiagen) with a mixture of one 2.8 mm ceramic (Qiagen), five 1 mm zirconia (BioSpec Products) and ~ 100 mg of 425–600 μm glass beads, acid washed (Sigma-Aldrich) in 50 µl of nuclease-free water (Sigma-Aldrich). Homogenization was carried out in two steps (2 × 2 min at 30 Hz). After the first 2 min the tube racks were rotated to ensure uniform disruption and homogenization of all samples. In case the samples were not uniformly crushed yet, the homogenization was repeated. DNA extraction was performed using the DNeasy 96 Blood & Tissue Kit (Qiagen) following the manufacturer’s instructions with a final elution volume of 50 µl. As the used *M. robertsii* strain (ARSEF 2575) is fluorescently labelled, with each spore carrying a single copy of the *mRFP1* gene [77], we could determine absolute spore numbers of our samples by targeting the *mRFP1* gene. Primers and probe were designed to bind to the *mRFP1* gene (gene bank accession number: KX176868.1;

*mRFP1*_Forward: 5’-CTGTCCCCTCAGTTCCAGTA, *mRFP1*_Reverse: 5’- CCGTCCTCGAAGTTCATCAC, *mRFP1*_probe: 5’[6FAM]AGCACCCCGCCGACATCCCCG[BHQ1]) using Primer3Plus software [83] and were confirmed to only amplify the gene of interest.

The ddPCR reactions with a total volume of 22 µl were prepared as follows: 11 µl of 2x ddPCR Supermix for Probes (Bio-Rad), 900 nM of both primers (Sigma-Aldrich) and 250 nM probe (Sigma-Aldrich), 10 U of both enzymes EcoRI-HF and HindIII-HF (New England Biolabs), 5.27 µl nuclease-free water (Sigma-Aldrich) and 2.2 µl of template DNA. Droplets were generated according to manufacturer’s instructions with the QX200 Droplet Generator (Bio-Rad). The amplification program was initiated with a first step at 95 °C for 10 min to activate the DNA polymerase, followed by 40 cycles of 30 s at 94 °C and 60 s at 56 °C, and finished with 98 °C for 10 min to stop the reaction. For the entire program the ramp rate was set to 2 °C/sec. After amplification droplets were analysed on the QX200 Droplet Reader (Bio-Rad) for the readout of positive and negative droplets.

Data analysis was done using the QuantaSoft Analysis Pro Software (Version 1.0.596; Bio-Rad). The threshold was manually set to 3000. Overloaded samples (low amount of negative droplets) were diluted and re-run. Each run included a H_2_O negative control (which in 2 of 6 cases showed a single positive droplet). We therefore only considered males that had at least 2 positive droplets in the PCR to be above detection threshold and to have confirmed spore load (either being the exposed male or by cross-contamination from the exposed male). In 8/48 fights we were not able to differentiate between the healthy and infected male as the fungal loads of both males were below detection threshold. These fights could thus not be included in analyses, in which identity of the males was required.

### Observation of male and worker aggression

We quantified male and worker aggressive actions by behavioural scan sampling for 97 high-dose and 111 low-dose fights (including their respective control fights of two healthy males, see Table S1; all of these fights included colour-coded males, total N = 208 observed fights). Observers were blind for both fight combination (healthy-healthy vs. healthy-infected) and individual male treatment (colour code did not reveal treatment). A total of 18 scans (each taking one to several seconds) were performed per fight, one every 30 min for the first 8 h and one last one after 24 h, when we also determined fight outcome (note that some fights were still undecided after 24 h). For each male, we determined the number of aggressive interactions performed towards the other male, which included several behaviours like *biting*, *holding* (grabbing the rival with the mandibles) and *besmearing* (male bending its gaster tip to apply hindgut secretion onto the rival). When one of the behaviours occurred alone, the aggression score of the respective scan was 1, when two categories were performed simultaneously, like e.g. holding and besmearing, both were counted, leading to an aggressive score of 2 for that scan. We also counted the number of aggressive interactions that workers performed towards either of the males. Worker aggression included *biting*, *carrying* (male is held with the mandibles and carried around), *dragging* (male is held and pulled over the ground), and *dismembering* (body parts are intensively attacked or already bitten off). As in the case of male aggression, we counted each behavioural category as one aggression (independently of how many workers performed the same behaviour), leading to aggression scores of > 1 when multiple behavioural categories were performed at the same time (e.g. biting by one and dragging by a second worker). For each male, we then summed over all 18 scans to receive one total score of the aggressive acts it performed towards the other male and one for the aggressive acts it received by the workers.

### Determination of male mortality risk in the absence of fighting

To determine the *baseline mortality* of the infected males (and their healthy controls) in the absence of fighting, we kept males of each infection stage and level with five of its colony’s workers, but no second male, for 24 h and determined their survival. We used a total of 268 males (113 control and 115 infected, as detailed in Table S3).

### Activation of the immune system in mild and severe infection

We determined the immune gene activation that males show in the middle and at the end of the fighting period for 57 additional colour-marked males following treatment with the sham solution, the low or the high pathogen dose, and keeping them individually for 12 or 24 h after exposure (N = 9–10 males per time point and dose combination, see figure legends of Fig. 2, S2 for details). For each male, we determined the expression levels of three different immune genes, involved in the cellular and humoral immune response against fungal infection: (i) Relish (*Rel*) – a transcription factor in the IMD pathway [53, 84], (ii) the shared homolog of the Melanisation Protease 1 and 2 (*MP1* and *MP2*) from *Drosophila melanogaster*, and the Prophenoloxidase-activating enzyme (*PPOAE*) from *D. mauritiana*, which we refer to as Prophenoloxidase-activating factor (*PPOAF*), a gene that is involved in melanisation as it activates *PPO* [55–57], and (iii) the antimicrobial peptide Defensin (*Def*) that is activated by the Toll-pathway and destroys fungal cell walls [58, 59]. We used the elongation factor 1-alpha (*EF1A*) as housekeeping gene [85].

Males were individually snap-frozen in 1.5 ml Safe-Lock Tubes (Eppendorf), their RNA extracted and transcribed to cDNA before performing gene expression analysis using ddPCR. Total RNA was extracted using the Maxwell® RSC instrument together with the Maxwell® simplyRNA Tissue Kit (Promega) according to the manufacturer’s instructions with a final elution volume of 60 µl. Prior to RNA extraction individuals were homogenized in 200 µl homogenization buffer including 4 µl 1-Thioglycerol using a TissueLyser II (Qiagen) with a mixture of five 1 mm zirconia (BioSpec Products) and ~ 100 mg of 425–600 μm glass beads, acid washed (Sigma-Aldrich). Homogenization was carried out in two steps (2 × 2 min at 30 Hz). After the first 2 min the tube racks were rotated to ensure uniform disruption and homogenization of all samples. In case the samples were not uniformly crushed yet, the homogenization was repeated. To ensure the complete removal of residual DNA contamination an additional DNase-I treatment (Sigma-Aldrich) step was performed prior to the reverse transcription. The cDNA synthesis was performed using the iScript cDNA synthesis kit (Bio-Rad) according to manufacturer’s instructions.

To analyse the expression patterns of the immune genes, we used multiplex ddPCR assays, each targeting one immune gene and the housekeeping gene. The *EF1A* sequence was identified using the primer sequences published in [85]. To identify the target sequences of the immune genes of interest in *C. obscurior*, as a first step well-annotated protein sequences of *Drosophila* (*Rel* - NP_477094.1, *PPOAF* - XP_033167441, *Def* - NP_523672.1) were used to identify the respective protein sequences in the two ant species *Atta cephalotes* (Acep) and *Temnothorax longispinosus* (Tlon). These two species were chosen because of the annotation status of their genomes and their phylogenetic similarity to *C. obscurior*. The thereby identified sequences of *A. cephalotes* (Acep; *Rel* - XP_012059341.1, *PPOAF* - XP_012057242.1, *Def* - XP_012058992.1) and *T. longispinosus* (Tlon; *Rel* - TGZ54393.1, *PPOAF* - TGZ55126.1, *Def* - TGZ47143.1) were blasted against the *Cardiocondyla obscurior* (Cobs) genome [86], accessible under GenBank number GCA_019399895.1 (*Rel* - COBS06226, *PPOAF* - COBS07801, *Def* - COS15672). Primers were designed using *Primer3Plus* [83]) and *Multiple Primer Analyzer* (https://www.thermofisher.com) software. The following primers and probes were used for the respective genes: *EF1A*_Forward: 5’- ATTGGAACAGTACCCGTTGG, *EF1A*_Reverse: 5’-CACCCTTCGGTGGGTTATTT, *EF1A*_probe: 5’[HEX]ACCTGGTATGGTCGTTACCTTTGCACCCGT[BHQ1]; Relish: *Rel*_Forward: 5’-ACGGATTTAGGATGGACACC, *Rel*_Reverse: 5’- TTGGTGGCTTCCTTCAACA, *Rel*_probe: 5’[6FAM]TGCTCTCTTGTGCAGACTGGCGCAGA[BHQ1]; *PPOAF*: *PPOAF*_Forward: 5’- TGCTGCTCACTGTATCAAGG, *PPOAF*_*Reverse*: 5’- TCTGTTTCAGTGTCGGTGTC, *PPOAF*_probe: 5’ [6FAM]ACTGGCGTCTGACCAGCGTCCGT[BHQ1]; Defensin: *Def*_Forward: 5’- ACGGGCCTACTTACGAATTG, *Def*_Reverse: 5’- CGCAAGCACTATGGTTGATG, *Def*_probe: 5’[6FAM]CGAAGAGGAGCCGTCACACCTGACGC[BHQ1].

The ddPCR reactions with a total volume of 22 µl were prepared as follows: 11 µl of 2x ddPCR Supermix for Probes (Bio-Rad), 900 nM of each primer (Sigma-Aldrich) for the immune gene of interest as well as the housekeeping gene and 250 nM of the respective probes (Sigma-Aldrich), 3.74 µl nuclease-free water (Sigma-Aldrich) and 2.2 µl of cDNA. The cDNA was sonicated (15 s) before being added to the reagent mix. All further steps were performed as described above, except that the amplification program was slightly modified to improve the separation between positive and negative droplets. The amplification program was initiated with a first step at 95 °C for 10 min, followed by 50 cycles of 30 s at 94 °C and 2 min at 56 °C, and finished with 98 °C for 10 min. All PCR steps were carried out with a ramp rate of 1 °C/sec. The thresholds were set manually as follows: *EF1A* 4000, *PPOAF* 1500, *Rel* 2000 and *Def* 4000. Immune gene expression levels were normalized to the reference gene *EF1A*.

### Statistical analysis

All statistical analyses were performed in the program ‘R’ version 4.0.3 [87]. We tested the difference between the two fight combinations (healthy-healthy vs healthy-infected) separately for each scenario (early stage low and high dose, late stage high and low dose). This was both due to (i) our experimental design, as a 48 h isolation period was required in the late but not the early infection treatment, and (ii) experimental constraints, since males only emerged from their colonies over multiple months, given that 694 males were required for the 347 fights, another 268 for the determination of male mortality risk in the absence of fighting and further 57 for the immune gene expression, totalling to 1019 males. We could therefore run simultaneous experiments of healthy-healthy and healthy-infected combinations of the same infection stage and dose, but not across all treatments. For statistical analysis, we applied Chi-Square tests (or Fisher’s exact tests when expected frequencies were < 5) or generalized mixed models (GLMM) (‘lme 4’ [88]). We assessed model assumptions (residual normality and heterogeneity and no overdispersion) with ‘DHARMa’ [89] and checked model stability and the presence of influential data points (Cook’s distance and dfbetas). Whenever multiple inferences were made from the same dataset, we corrected the overall p-values for multiple testing with the Benjamini-Hochberg procedure to protect against a false discovery rate of 0.05 [90]. We report two-sided, corrected p-values. All graphs were made using the ‘ggplot2’ package [91].

#### Fight outcome

To determine whether the fight outcome (one male dead, both males dead, or both males alive) differed between fight combinations (healthy-healthy vs. healthy-infected) for each scenario (early stage low and high dose, as well as late stage high and low dose), we ran logistic regressions with binomial error term and logit-link function for each outcome and implemented fight outcome as response variable and fight combination as predictor variable (multiple testing adjustment for 3 comparisons each; Fig. 1a,b, Table S2) and included ‘colony’ as a random effect. To further test for the decided fights whether early- or late-stage infected males were losing more often against the healthy rival than expected by chance, we used Chi-Square tests or, if the minimum expected frequency was less than 5, Fisher’s exact tests (Fig. 1a,b).

#### Male aggression performed towards its rival

For each of the 112 observed fights with one healthy and one infected male (see Table S1), we summed the number of male aggressive behaviours observed against its rival as detailed above and analysed them as count data. For each fight scenario (early stage low and high dose, late stage high and low dose), we ran GLMMs with negative binomial error term and log-link function as the data was over-dispersed, with male infection status (healthy vs. infected) as predictor and the number of male aggressive acts as response. ‘Colony’ and ‘fight replicate’ were included as random effects (multiple testing adjustment for 2 comparisons each). While the statistics are performed with the raw data, we visualise the infected male’s aggression in comparison to its healthy rival (by subtracting the infected male’s aggression score from that of the healthy male for each fight; Fig. 1c,d). We ran the same models also for the control fights between the two healthy males (N = 46 early and N = 50 late control fights, Table S1; low and high doses pooled, as both males in the control fights had only received sham treatment across dosages), by randomly assigning the two males per fight into either male A or male B, and iterating the model 100 times. None of the models showed a significant difference between male aggression of males categorized randomly into A and B males, confirming that male aggression was also balanced in the control fights.

#### Worker aggression towards males

For each of the 112 observed fights with one healthy and one infected male we further summed the number of worker aggressive acts against each male as detailed above (count data). To test if the infected vs. healthy males received a different level of aggression by the workers, we ran GLMMs with Poisson error terms and log-link function with male infection status (healthy vs. infected) as predictor and the number of worker attacks as response. ‘Colony’ and ‘fight replicate’ were included as random effects (multiple testing adjustment for 2 comparisons each). Statistics were run on the raw data, while we visualise the difference of worker aggression towards the infected males compared to that towards the healthy male of the same fight by subtracting the aggression towards the infected male from that directed towards the healthy male (Fig. 1e,f). Again, we ran the same models also for the 46 early and 50 late control fights, with randomized categorisation of the two males per fight into male A or B, and 100 times iteration of running the models, and corrected for multiple testing. For the early fights, 2/100 iterations showed a significant difference between worker aggression received by male A vs. B. In the late fights, 16/100 iterations showed imbalanced worker aggression, which was mostly due to one influential fight, in which workers aggressed one of the males –the later loser– 21-fold more than in the other control fights (15 attacks vs. a mean ± SD of 0.69 ± 1.36 attacks per male in all remaining late control fights ), with always the category A or B being more aggressed to which this highly-attacked male was randomly assigned to (in our set of iterations 41% A and 59% B). Exclusion of this special fight led to only 7/100 of the iterations showing significant imbalance of worker aggression. Similar to fights between differentially-aged males [40], over-proportional worker aggression against the later loser was therefore also possible in fights between the two similarly-aged healthy males in our experiment, yet without any predictable pattern between males A and B.

#### Male mortality in the absence of fighting

To determine if the baseline risk of the males to die during the 24 h fight period in the absence of fighting was higher for the infected males than their respective healthy male control, we ran a logistic regression with binomial error term and logit-link function with male survival (dead vs. alive) as response and male infection status (healthy vs. infected) as predictor for each dose and stage of infection (Fig. 1 g,h, S1; Table S3).

#### Costs of fighting

To determine the additional costs of fighting of infected males, we compared the survival of non-fighters (baseline risk) to the survival of fighting males for each dose and infection stage. We considered fights with one healthy and one infected male that either ended decided (one dead) or both males being dead and calculated the proportion in which the infected males were dying and compared it to the proportion of male mortality in the absence of fighting using Chi-Square tests or, if the minimum expected frequency was less than 5, Fisher’s exact tests (Fig. 1 g,h; multiple testing adjustment for 4 comparisons each).

#### Immune gene activation

To test whether the gene expression differed between infected and healthy males at 12 and 24 h after exposure for both the low and high dose, we compared their normalised gene expression values (value of immune gene / value of housekeeping gene) by Wilcoxon-tests for independent samples (multiple testing adjustment for 6 comparisons). For visualisation (Fig. 2, S2), we show the difference of the infected males to their respective healthy male control, by subtracting the median of the healthy males from each infected male.

### Electronic supplementary material

Below is the link to the electronic supplementary material.

Supplementary Material 1

Supplementary Material 2

Supplementary Material 3

## Data Availability

The datasets generated and analysed during the current study are available in the Dryad data repository; 10.5061/dryad.bk3j9kdhb.
