# Supplementary material for: Trade-offs between immunity and competitive ability in fighting ant males
Source: BMC Ecol Evol. 2023 Aug 7;23:37. doi: 10.1186/s12862-023-02137-7 (PMC10405452; doi:10.1186/s12862-023-02137-7)
Supplement: Supplementary file 1 — Supplementary Material 1 [file 12862_2023_2137_MOESM1_ESM.pdf]

Supporting Figure 1

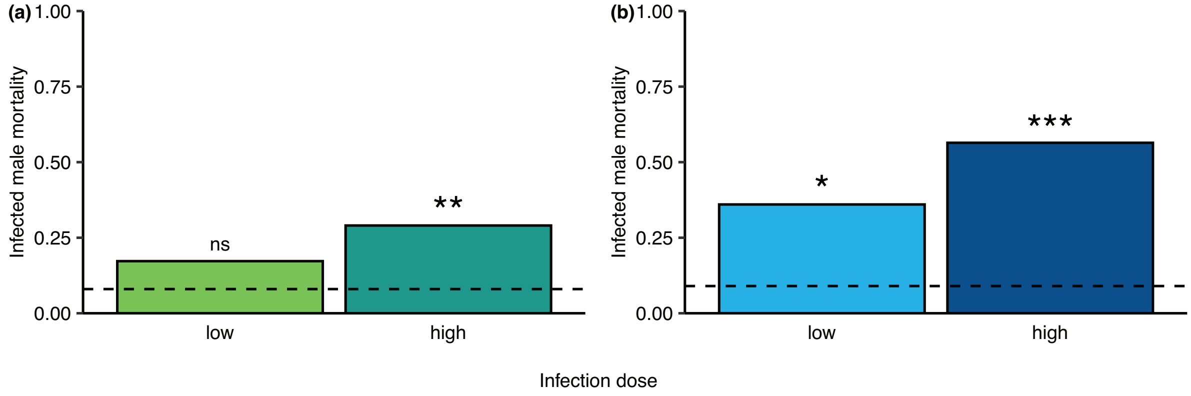

Figure S1) Baseline mortality risk of males in the absence of a fight in dependence of infection dose and stage. At the early **(a;** green**)** and late **(b;** blue**)** stage of infection, the risk for an infected male to die above the risk for healthy males (dotted line) is shown for the low and high dose (darker tone reflects higher dose). Infected male mortality was significantly increased for all males except when in the early stage of infection with a low dose. Males at the late stage of a severe infection were highly moribund, not surviving the 24h in >50% even without fighting. Based on 268 males. Significant deviation to healthy male group indicated by ** $p<0.01$, *** $p<0.001$, ns=non-significant.
