# Supplementary material for: Trade-offs between immunity and competitive ability in fighting ant males
Source: BMC Ecol Evol. 2023 Aug 7;23:37. doi: 10.1186/s12862-023-02137-7 (PMC10405452; doi:10.1186/s12862-023-02137-7)
Supplement: Supplementary file 2 — Supplementary Material 2 [file 12862_2023_2137_MOESM2_ESM.pdf]

Supporting Figure 2

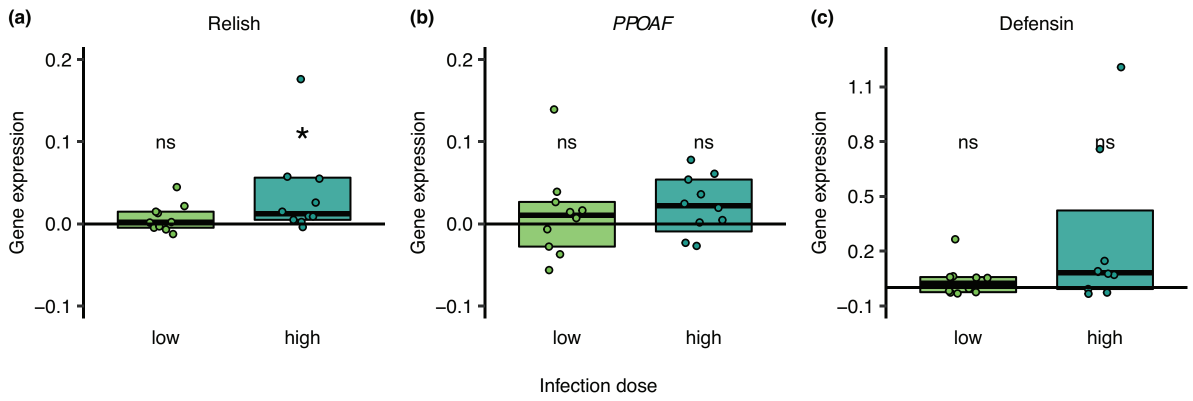

Figure S2) Immune gene expression 12h after exposure to the low or high pathogen dose. Expression of infected males' immune genes (**a**) Relish, (**b**) *PPOAF*, and (**c**) Defensin showed no significant activation 12h after exposure over the baseline expression of the healthy males (zero line), except for the transcription factor Relish after exposure to the high pathogen level. For each infected male we show its relative gene expression level normalised to the housekeeping gene *EF1*, relative to the median gene expression of the respective healthy control males (zero line) as individual data point, the group's median (black line) and 95% CI (box). Based on a total of 29 males (N=9 control males, 10 low-dose and 10 high-dose exposed). Significant deviation to the healthy males for each dose and gene shown by * $p<0.05$, ns=non-significant.
