# Supplementary material for: Trade-offs between immunity and competitive ability in fighting ant males
Source: BMC Ecol Evol. 2023 Aug 7;23:37. doi: 10.1186/s12862-023-02137-7 (PMC10405452; doi:10.1186/s12862-023-02137-7)
Supplement: Supplementary file 3 — Supplementary Material 3 [file 12862_2023_2137_MOESM3_ESM.pdf]

Supporting Tables

Table S1) Number of fights per infection dose and stage. Sample size for all fights between a healthy male and a male exposed to the low or high pathogen dose, and their respective control fights between two healthy males. Fight outcome after 24h was determined for all 347 fights. 97 out of the 236 high-dose fights and all 111 low-dose fights were further observed for detailed behavioural interactions between the two males and the workers towards the males, for which both males were individually colour-coded before the fight. 139 high-dose fights were analysed only for fight outcome, so that males remained unmarked males during the fight (with the exception of 12 fights, in which males were colour-coded but only fight outcome was analysed), and their identity later determined by spore-load PCR for the 48 (20 early stage and 28 late stage fights) out of the 77 fights between a healthy and an infected male that had a clear winner and a clear looser (i.e. were 'decided'). Note that the classification of the males into the infected vs healthy male based on spore load, was only possible in 40/48 fights, see methods, and that the method is not sensitive enough for the low load fights).

Infection stage	Dose	Fight combination	# Fights outcome only	# Fights behaviour & outcome	# Fights total
early	low	healthy-healthy	0	30	30
		healthy-infected	0	30	30
	high	healthy-healthy	27	16	43
		healthy-infected	48	17	65
late	low	healthy-healthy	0	20	20
		healthy-infected	0	31	31
	high	healthy-healthy	23	30	53
		healthy-infected	41	34	75

Table S2) Statistical results of fight outcome depending on infection dose and stage. For each fight (Table S1), it was determined if both males were still alive or had died during combat, or if there was a clear winner or loser (one male alive, the other dead; numbers of fights per outcome given). Sample size, test statistics and p values provided for the GLMMs between the control fights of two healthy males and fights between one infected and one healthy male. All df=1. Significant and trending p values indicated in bold (pie chart inserts in Figs.1a,b). The last column shows in how many of the decided fights between an infected and a healthy male (number and % of fights) the infected male was the loser (bars in Figs. 1a,b; statistical results reported in the main file). Value in brackets indicates the number of fights in which we were able to clearly differentiate between the males.

**Infection stage**	**Dose**	**Fight outcome**						
		**both alive**		**both dead**		**winner/loser**		
		**healthy-healthy**	**healthy-infected**	**healthy-healthy**	**healthy-infected**	**healthy-healthy**	**healthy-infected**	**Infected male losing**
early	low	14	20	1	0	15	10	4
		χ^2=2.462, p=0.236		χ^2=1.462, p=0.236		χ^2=1.724, p=0.236		40%
	high	21	17	4	8	18	40	34
		χ^2=5.994, **p=0.043**		χ^2=0.241, p=0.623		χ^2=4.258, **p=0.059**		85%
late	low	10	16	2	7	8	8	3
		χ^2=0.013, p=0.910		χ^2=1.411, p=0.433		χ^2=1.125, p=0.433		37.5%
	high	22	10	3	11	28	54 (46)	43
		χ^2=13,119, **p<0.001**		χ^2=2.710, p=0.100		χ^2=4.936, **p=0.039**		93.5%

Table S3) Baseline mortality of males in the absence of fighting depending on infection dose and stage. Sample size of males that were observed for their mortality in the absence of fighting during the 24h period immediately after exposure (early infection stage) or 48h post exposure (late infection stage), and number of males dying during this period. See Figs. S1a,b.

		healthy		infected	
**Infection stage**	**Dose**	**N observed**	**N died**	**N observed**	**N died**
early	low	60	5	29	5
	high			62	18
late	low	53	5	25	9
	high			39	22
